# Primary non-Hodgkin's breast lymphoma: Surgical approach

**DOI:** 10.1186/1757-1626-1-311

**Published:** 2008-11-14

**Authors:** Alessandro Neri, Stefano Caruso, Guido Cerullo, Maria P Lenoci, Daniele Marrelli, Franco Roviello

**Affiliations:** 1 Department of Human Pathology and Oncology – Section of General Surgery and Surgical Oncology, Division of Hematology, University of Siena, viale Bracci-Policlinico "Le Scotte" 53100, Siena, Italy

## Abstract

We report the case of a 38-year old woman affected by primary lymphoma of the right breast, with disease progression after chemotherapy and subsequent radiotherapy, successfully treated with a modified radical mastectomy. The literature of primary breast lymphomas has been reviewed and discussed in relation to our case. Our experience stresses the importance of a radical surgical approach in a locally advanced non-Hodgkin's lymphoma of the breast unresponsive to radio and chemotherapy.

## Background

Primary lymphoma of the breast (PBL) is a rare disease and all published series include a small number of patients. We present the case of a 38-year old woman with a primary non-Hodgkin lymphoma of the right breast who was unsuccessfully treated by primary chemo and radiotherapy. The patient was then referred to our Department of General Surgery and Surgical Oncology and treated with a modified radical mastectomy which allowed complete removal of the tumor and local control of the disease.

We report this case of a locally advanced PBL not responsive to chemo and radiotherapy in order to emphasize the role of the surgical approach in this subset of patients.

## Case presentation

**Figure 1:**
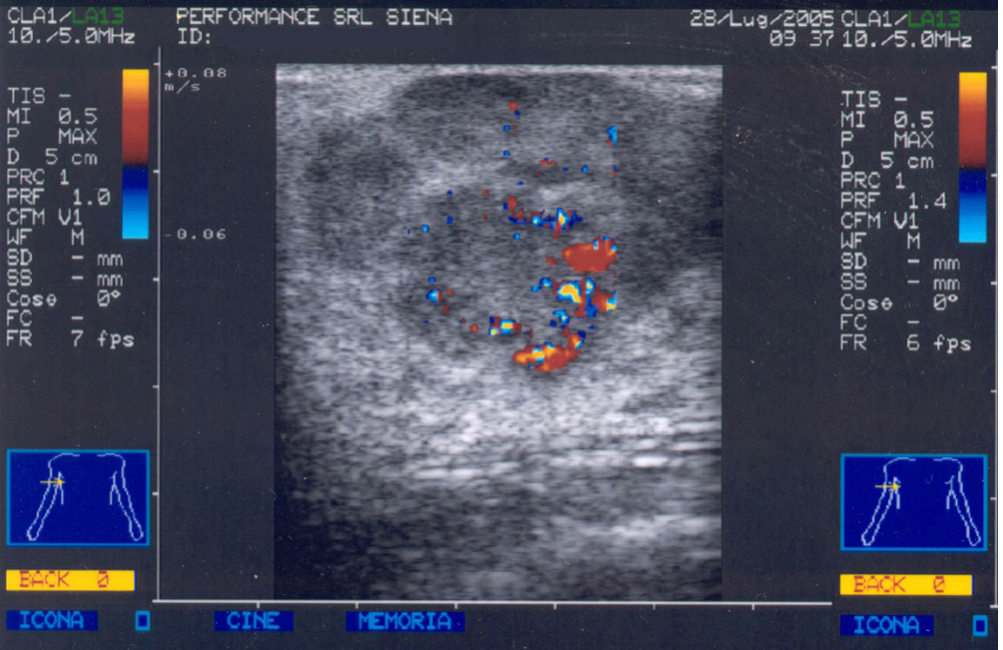
Breast ultrasonography (US) shows the presence of a hypervascularized non-homogeneous mass with ill-defined margins.

**Figure 2:**
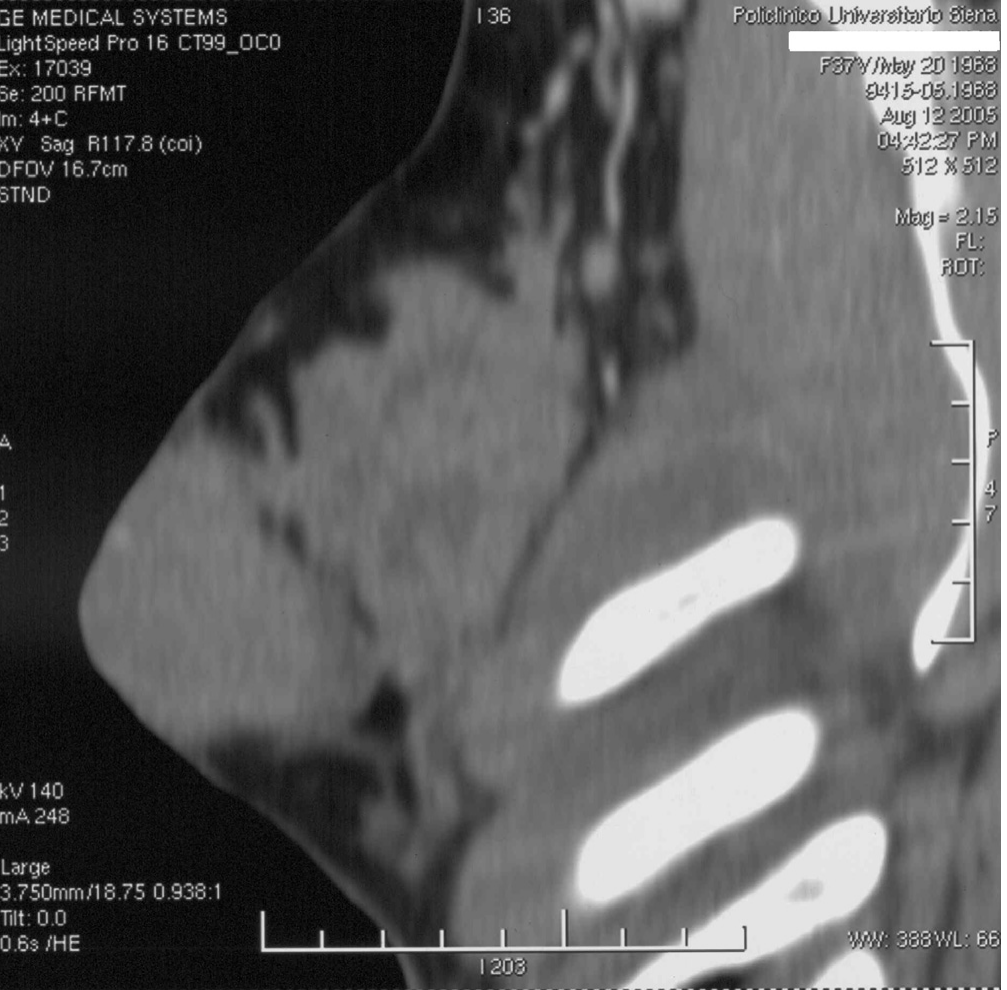
A CT scan shows a large breast mass associated with pathological enlarged lymph nodes in the right axilla.

A 38-year-old woman was admitted to our hospital in July 2004 presenting a right breast mass. A physical examination revealed an elastic and mobile mass occupying the upper quadrants of the right breast. Examination of the axilla and neck was negative for enlarged lymph nodes. Mammography showed a large mass of the right breast with multiple opacities in its contest. Breast ultrasonography (US) confirmed the presence of a hypervascularized non-homogeneous mass with ill-defined margins (Figure 1). A percutaneous aspiration citology (FNAC) was performed but resulted negative for malignant cells. The mass showed a rapid growth and a second FNAC diagnosed a diffuse large B-cell lymphoma (DLBCL) with a proliferation rate of more than 90%. Immunostaining profile by monoclonal and polyclonal antibodies showed positivity for CD5, CD20, Bcl 2, Bcl 6 and MUM1 and absence of staining for CD10 and Cycline D. A thoracic and abdominal computed tomography (CT-scan) confirmed the presence of the large breast mass associated with pathological enlarged lymph nodes in the right axilla (Figure 2). A bone-marrow biopsy resulted negative for lymphomatous infiltration. The patient was diagnosed as stage I_E _according to the Ann Arbor staging system and it was graded into the low risk group with an IPI score of 1 [1,2].

PBL is considered a disease with potential systemic diffusion, to which primary chemo and radiotherapy are presently considered the most effective therapeutic approach. Since the addition of Rituximab (R), a monoclonal antibody anti-CD20, showed an improvement of results in the first line treatment of DLBCL, we treated the patient with the immunochemiotherapy schedule R-CHOP (*Rituximab *375 mg/m2, *Cyclophosphamide *750 mg/m2, *Vincristine *1,4 mg/m2, *Doxorubicin *50 mg/m2, *Prednisone *60 mg/m2), recycled every 3 weeks for 6 cycles. Although a CT scan after the third cycle demonstrated a reduction of the tumour mass of about 85%, which remained stable until the end of the chemotherapy, a rapid growth of the residual mass was showed already one month later.

**Figure 3:**
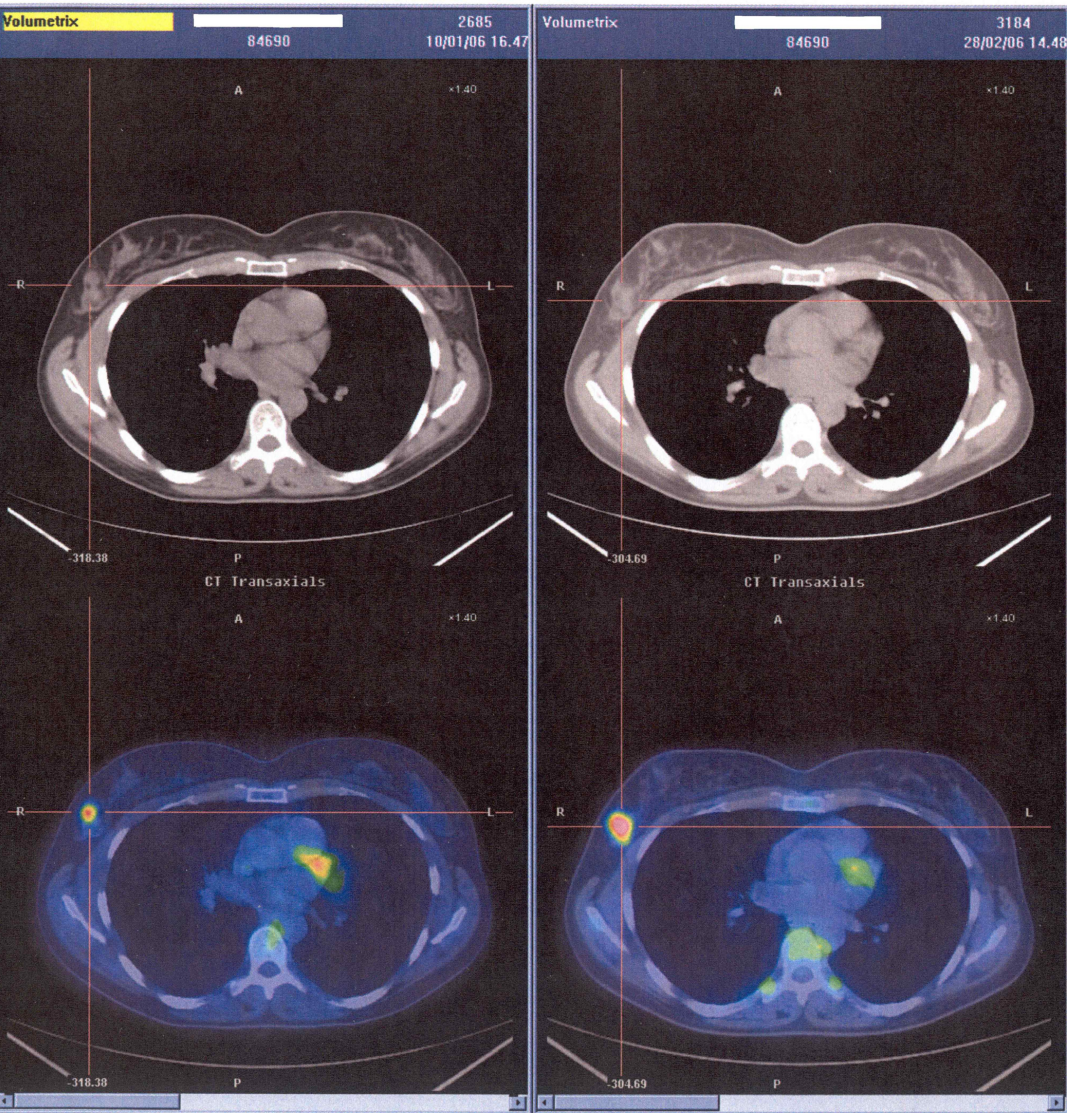
A PET-CT shows a local relapse of disease.

A second-line polichemotherapy with HAM schedule (high dose of *Cytosine Arabinoside *6 g/m^2 ^and *Mitoxantrone *12 mg/m^2^) was then performed in January 2005. However, the breast lesion showed further progression one month after. The systemic staging of disease by PET-TC excluded distant localizations of the disease. In March 2005 the patient received radiotherapy (44 Gy) on the right breast and omolateral axilla, obtaining disease stabilization, but local relapse was noted 3 months later and a PET/CT scan demonstrated enlarged right axillary lymph nodes (Figure 3).

The patient was then referred to our Department for surgical evaluation. Despite the breast mass occupied whole the upper and external quadrants of the right breast, it was mobile on the underlying muscular layers. Enlarged lymph nodes were palpable in the right axilla. We therefore performed a right modified radical mastectomy, with sparing of the pectoral muscles, completed with a level III axillary lymphadenectomy in order to remove the enlarged axillary lymph nodes.

The surgical specimen was examinated by an expert pathologist and the histological response confirmed the diagnosis of DLBCL without skin, nipple, areola and muscle wrap involvement. Seventeen axillary lymph nodes examinated were not involved by tumour cells. The postoperative course was uneventful and on second postoperative day the patient was discharged.

At present, four and three years respectively later the medical and surgical approach, the patient is disease free at clinical and instrumental follow up.

## Discussion

Non-Hodgkin lymphomas of the breast are uncommon cancers which occur either as primary extranodal diseases or as secondary localizations of a systemic disease. PBLs have a reported incidence ranging from 0.04% to 0.5% of all breast malignancies [3,4]. PBLs account for less than 1% of all patients with non-Hodgkin's lymphomas and approximately for 1.7% of all extranodal non-Hodgkin's lymphomas [3,4].

The criteria for the diagnosis of primary breast lymphoma were suggested by Wiseman and Liao in 1972 and include: (a) adequate pathologic evaluation, (b) close association between lymphomatous infiltrate and mammary tissue and (c) exclusion of either systemic lymphoma or extramammary lymphoma, except simultaneous ipsilateral axillary node involvement [5]. Patients with breast involvement as a result of progression or relapse of a previously diagnosed non-Hodgkin's lymphoma are considered as secondary breast lymphomas.

Available information regarding PBL are difficult to analyze and to compare because of the relatively small number of patients in most series and the substantial differences in criterions of classification that has been used, such as lymphoma subtypes, histopatologic terminology, treatment modalities and staging systems. Moreover, some among larger reports have also included secondary breast lymphoma [4]. Overall, the characteristics of this disease, such as natural history, prognostic factors and impact of treatment have not been yet well established.

Most studies reported a preponderance of right-sided presentation in breast lymphoma, but others reported the opposite [6,7]. Bilaterality has been reported to occur in 1% to 14% of cases [6]. The incidence of breast lymphoma in men is extremely low [8].

Breast lymphoma and carcinoma usually have a similar clinical and radiological presentation. Patients presenting such of these malignancies generally refer to physician for a painless enlarging breast mass.

Both lymphoma and carcinoma of the breast are diseases of the middle-aged and elderly, although a small subset of lymphomas (Burkitt's or Burkitt-like lymphomas) presents at a young age, typically with bilateral involvement and rapid progression. The reported median age for breast lymphoma ranges from 51 to 60 years, but with a wide range [6,7].

Even if breast lymphomas generally present no calcifications on mammograms [9], at present there are no clinical or radiological findings which allow a differential diagnosis between lymphoma and carcinoma of the breast. The cornerstone of the diagnostic process remains tissue biopsy and histopathologic examination with appropriate immunophenotyping.

In our case the patient showed an atypical presentation with a clinical diffuse involvement of the right breast, probably due to coexisting mastitis.

In our experience, fine needle aspiration cytology (FNAC) resulted a reliable diagnostic technique, in accordance with the high sensitivity reported in literature ranging from 83% to 100% [10].

Diffuse large B-cell lymphoma (DLBCL) is the most common histological subtype, representing 45% to 79% of all PBL cases [6,7].

Reported 5-year overall survival rates have varied from 9% to 85% [6,11,12]. Tumor size, bilateral presentation and axillary lymph node involvement are reported not to affect the prognosis, while an important prognostic factor is the histologic type of PLB in association with its tumor grading and stage [6].

The therapeutic management is controversial and is not fully established as yet. The frequency of surgery has been declining since 1990 and this approach is presently not indicated neither in case of lesions unresponsive to chemo and radiotherapy nor for relapsing disease. Surgical treatments are seldom reported in literature, although radical approaches have been abandoned on behalf of less invasive treatments [13,14].

Combined radiation and chemotherapy seems to be the most effective treatment, even in the early stages of PBL [6,13,14]. Most authors suggest that chemotherapy and radiotherapy should be the initial, and in most cases the only treatment. Indeed, the number of patients successfully treated by a combination of chemotherapy and radiation therapy has been increasing in recent years and this approach has been proposed as the gold standard treatment for PBL [6,13,14].

We reported a particular case of local advanced PLB that showed a very poor response to systemic therapy. In fact, our case had demonstrated a lack response either to the first line chemotherapy protocol and to subsequent salvage chemotherapy. The patient has been then unsuccessfully treated with radiotherapy, demonstrating further increase of the mass which virtually occupied the whole breast at the time of referral to our attention.

Surgical approach by radical mastectomy allowed removal of the lesion with free margins and resulted in optimal control of the disease, with the patient alive without relapse at 36 months from surgery. Axillary lymphadenectomy was associated because of lymph nodes clinically involved. However, in agreement with other authors [13], we do not consider axillary lymph nodes dissection necessary. Indeed, the reported incidence of involvement of the lymph nodes among PBL patients ranges between 40 and 50% [13], but there is no evidence that axillary clearance may improve the long term outcome.

Even if several authors argue that mastectomy is not necessary and most advocate systemic chemotherapy as the proper treatment, further investigation on PLB is required to define reliable prognostic factors and optimal management and based on our experience we suggest that radical surgery is an effective and safe option in those cases not responsive to chemo-radiotherapy.

## Consent

Written informed consent was obtained from the patient for publication of this case report and accompanying images. A copy of the written consent is available for review by the Editor-in-Chief of this journal.

## Competing interests

The authors declare that they have no competing interests.

## Authors' contributions

We certify that all the authors have participated sufficiently in the work to take public responsibility for appropriate portions of the content. SC and GC gave substantial contributions to conception of manuscript, contributing to design and acquisition of data, and reviewing the literature. MPL has followed the patients during the oncologic treatment and contributed to the layout of the manuscript content related to this treatment, in critical relationship to the most recent evidence based medicine. AN drafted the manuscript in the final actual form, participating to analysis and interpretation of data. DM and FR have been involved in drafting the manuscript and revising it critically for important intellectual content. All authors read and approved the final manuscript.
